# Prevalence and correlates of cannabis abuse among residents in the community of Fort McMurray, a city in Northern Alberta which had endured multiple natural disasters

**DOI:** 10.3389/fpsyt.2022.962169

**Published:** 2022-09-21

**Authors:** Gloria Obuobi-Donkor, Ejemai Eboreime, Reham Shalaby, Belinda Agyapong, Vincent I. O. Agyapong

**Affiliations:** ^1^Department of Psychiatry, University of Alberta, Edmonton, AB, Canada; ^2^Global Psychological E-Health Foundation, Edmonton, AB, Canada; ^3^Department of Psychiatry, Dalhousie University, Halifax, NS, Canada

**Keywords:** cannabis abuse, depression, anxiety, Fort McMurray, correlates

## Abstract

**Background:**

Cannabis is one of the widely used drugs for relaxation and may be abuse among users. Researchers have given attention to cannabis use among the general population while vulnerable populations who have experience multiple traumas may be at risk of cannabis abuse. Other factors may influence cannabis abuse among people exposed to traumas.

**Objective:**

The study aims to determine the prevalence and correlates of self-reported cannabis abuse among residents of Fort McMurray, a city in Northern Alberta, Canada.

**Methods:**

A cross-sectional survey was conducted using an online questionnaire. Sociodemographic data, trauma exposure, and clinical characteristics were collected to identify the possible risk factor of cannabis abuse. Data were analyzed with SPSS version 25 using a chi-square test and binary logistic regression analysis. Correlation analysis was also performed to ascertain likely cannabis abuse and the association with other mental health conditions.

**Results:**

Overall, participants who completed the survey were one hundred and eighty-sixed out of the two hundred and forty-nine residents who accessed the online survey, giving a response rate of 74.7%. The prevalence of self-reported cannabis abuse among participants was 14%. Most of the participants were females (159, 85.5%), more than 40 years of age (98, 52.7%), employed (175, 94.1%), owned their house (145, 78.0%), and 103 (60.6%) reported being exposed to at least a trauma (COVID-19, flooding, or wildfire). Regarding regression analysis results, participants living in a rented accommodation were nearly four times more likely to abuse cannabis (OR = 3.86; 95% CI: 1.34–11.14) than those owning their houses. Similarly, male participants were more than 6-folds more likely to abuse cannabis than the female gender (OR= 6.25; 95% CI: 1.89–20), and participants in a relationship were six times more likely to abuse cannabis than participants not in a relationship (OR = 6.33; 95% CI: 1.67–24.39). The study also found significant association of depressive symptoms (*X*^2^ = 4.561; *p* = 0.033) and anxiety symptoms (*X*^2^ = 4.700; *p* = 0.030) with likely cannabis abuse.

**Conclusion:**

Demographic factors significantly predicted likely cannabis abuse in the Fort McMurray population, and cannabis abuse significantly correlated with presence of moderate to high anxiety and depression symptoms. It is essential to mobilized resources to support vulnerable communities and manage cannabis abuse.

## Introduction

Globally, cannabis is a habitually used psychoactive substance (1) and one of the most extensively used recreational drugs in Canada (2). According to the United Nations Office on Drugs and Crime, in 2019, the annual prevalence of cannabis use was 3.86% globally (3). Since the legalization of cannabis in Canada in 2018, the usage has increased by about 2% among people 15 years and older from 14.9 to 16.8% in 2019 (2, 4). Similarly, statistics from the National Survey on Drug Use and Health recorded the prevalence of cannabis use at 17.9% in 2020 (5). In a systematic review and meta-analysis, the prevalence of cannabis use disorder and cannabis abuse among people who use cannabis was recorded at 22 and 13%, respectively (6).

This suggests that as the overall prevalence increases, the risk for cannabis use disorder increases (5, 7, 8). Cannabis misuse can negatively affect one's mental health, especially for those with comorbid anxiety or depressive disorders and is associated with suicidal ideation (7). Furthermore, individuals with mental illnesses like depressive and anxiety disorders and post- traumatic stress disorder are more prone to problematic cannabis use than the general population (9).

Experiencing mental health disorders is likely to cause a surge in the abuse of cannabis (10). The US saw an increase in the prevalence of cannabis use among people with depression from 2005 to 2017 (11). Clinical practice and epidemiological studies have also proven that a history of depressive disorder predisposes an individual to cannabis use (12, 13) which may lead to abuse and later worsen their conditions (11, 14). For example, a study among 728,691 people reported that individuals experiencing depression are 18.8% as likely to abuse cannabis as the rest of the population (11).

In the United States, major depressive disorder is one of the commonest psychological illnesses, with an estimated lifetime prevalence of 17% (15). This history of depressive disorder increases the prevalence of cannabis use among patients with major depressive disorder. The literature on why individuals with mental health history abuse cannabis are limited and it is also unknown whether there is likely cannabis abuse among those with various history of mental health conditions (11).

Numerous explanations have been reported why cannabis abuse may be more eminent among people with mental health conditions than those without. For example, Gruber et al. suggested that cannabis possesses antidepressant effects; thereby, the depressive patient uses it to reduce depressive symptoms (16). Other research suggests that individuals may use cannabis to induce sleep among those with post-traumatic stress disorder (PTSD) (17) and reduce anxiety symptoms (18). Notwithstanding, other clinical associations and sociodemographic predictors remain unclear, and further research is needed to explore these factors. Other demographic parameters may be attributed to the use of cannabis. For example, a comparative study conducted by Van Etten and Anthony revealed that the male gender has the potential to venture into drug usage and desire to explore substances when the opportunity arises (19). On the contrary, the same research group found that females will instead progress, and abuse cannabis once exposed to the substance (19). Marital status has also become a predisposing factor to cannabis use. A critical review among adults in the 21st century revealed that being unmarried increases the individual's chances of using cannabis (20). The neighborhoods and housing status may influence cannabis use. For example, a survey among the youth suggests that there is a 30% likelihood that deteriorating neighborhoods expose the youth to cannabis (21). Similarly, homeless youth and those living in critical housing situations usually use cannabis (22).

Other studies have focused on the exposure to traumatic events and the usage of substances (e.g., cannabis) leading to substance abuse (23–25). Fort McMurray residents have been exposed to various traumatic events including wildfire, flooding and the recent global pandemic which may lead to cannabis usage (26–29). A study by Agyapong et al. reported that participants in a cross-sectional study were more likely to abuse substances after the wildfire in Fort McMurray (24). Other studies have also examined the association between substance use among individuals who have experienced traumatic events (30, 31). However, these reasons would not explain why there may be an increase in likely cannabis abuse among people in Fort McMurray at this time. Further research is needed to establish the correlation between cannabis use and other mental health conditions, even though few studies have found significant associations between problematic drug use and likely mental health conditions (PTSD, depression and anxiety) (25). Irrespective of the contributions made by various studies, important questions remain regarding the factors influencing likely cannabis abuse among a vulnerable population who have experienced multiple traumas. To bridge the gaps in knowledge, we sought to assess the risk and identify the predictors of cannabis abuse and the association between mental health conditions and likely cannabis abuse among residents of Fort McMurray, where residents have experienced multiple traumas.

## Methodology

### Study setting

Fort McMurray is the urban service area of the Regional Municipality of Wood Buffalo in Northern Alberta, Canada. Following the 2021 census, the population of Fort McMurray was 106,059 (32). The population consists of 52.6% males and females 47.4% in the municipality (32). The municipality is dominated by the youth, with 42.4% between the ages of 20 and 44. Residents of Fort McMurray have experienced a series of traumatic events in recent times, such as the global pandemic (COVID-19) (33), the 2016 wildfire that destroyed homes and evacuated many residents (24), and the 2020 flooding (34) that threatened lives and properties.

### Study design and institutional review board approval

The study was a cross-sectional survey design, with quantitative data collected through self-administered online questionnaires with the REDCap software (35). Data were collected between April 24 and June 2, 2021. Participants provided consent when they clicked on the survey link and submitted responses. This study was conducted per the University of Alberta Reviews and Ethics Board (Pro00066054).

### Outcome and measures

The primary outcome measure was to assess the likely cannabis abuse among participants, through a self-reported question; have you abused cannabis in the past year.

Other variables of mental health conditions in the study were measured, depressive symptoms, resilience, PTSD, anxiety symptoms, and suicidal ideation.

The Patient Health Questionnaire (PHQ-9) was used to measure the depressive symptoms of participants (36). The nine-item were measured on a four Likert scale. The PHQ-9 scale categorizes depression based on scores into none-minimal (0–4 points), mild (5–9 points), moderate (10–14 points), moderately severe (15–19 points), and severe (20–27 points) (36). The scores were further categorized into two categories: none to mild depression and moderate to severe depression. The reliability and validity of the tool have good psychometric properties, and the internal consistency of the PHQ-9 has been shown to be high (36).

The brief Resilience Scale (BRS) was used to assess participants' resilience or the ability to recover from stress. A score ranging from 1.00-to 2.99 indicates low resilience, while a score ranging from 3.00 to 5.00 indicates high to normal resilience (35). Regarding reliability and validity literature shows that the BRS has good internal consistency, with Cronbach alphas ranging from 0.80 to 0.90, and test–retest reliability coefficients for a two-week interval were fair (0.61 to 0.69).

The Post Traumatic Stress Disorder Checklist Civilian (PCL-C) was used to assess likely PTSD symptoms. The level of distress produced by each symptom is rated on a five Likert scale from 1(not at all) to 5 (extremely) and a score of 44 or more was classified as likely PTSD and a score below 44 as unlikely PTSD (37). The PCL-C demonstrated good internal consistency and retest reliability and favorable patterns of convergent and discriminant validity (38).

The Generalized Anxiety Disorder-7 (GAD-7) scale measured participants' likelihood of anxiety symptoms. The self-reported tool consists of seven items rated on a four Likert scale. The score was recategorized into a binary; low anxiety (score <10) and moderate to high anxiety (score of 10 or more). A more severe symptom means a higher score (0–21) (39). The internal consistency and test–retest reliability of the GAD-7 was good, and it also provided good criterion, construct, factorial, and procedural validity (40).

The survey included a question on likely suicidal ideation (the ninth question of the PHQ-9 scale) which asked whether participants had passive death wishes/thoughts of self-harm in the last 2 weeks. In addition, the survey contained questions related to participants' mental health and medication history, as well as exposure to multiple traumas, i.e., COVID-19, wildfires, and flooding.

### Data collection and statistical analysis

All participants were requested to complete questions related to demographic, clinical and multiple trauma exposures (COVID-19, flooding, or wildfire). The survey questions distributed were programmed into REDCap, an online survey program. Participants were offered to fill out the online survey forms at their convenience.

Demographic variables included gender, age, employment status, and housing status. Clinical variables included the history of mental health diagnosis and previous psychotropic medication use, mental health counseling history, and willingness to receive mental health counseling.

Finally, the data collection form was chosen based on a literature review of some predictive factors included in survey questions, including variables to assess the likelihood of other mental health conditions; PTSD, anxiety, depression, suicidal ideation, and resilience (9, 41, 42).

The data were analyzed using SPSS Version 25 (43). Demographic, clinical, and multiple trauma exposure variables were examined against relationship status. Demographic characteristics were presented as raw numbers and percentages.

We were interested in examining the different clinical factors that may, at length, lead to the outcome of likely cannabis abuse. Cross-tabular analyses using the Chi-square test explored the association between demographic, clinical, and trauma variables and the likelihood of cannabis abuse.

A logistic regression model was performed, including the variables which were statistically significant or nearly significant (*p* ≤ 0.1) to the likelihood of participants abusing cannabis, derived from the Chi-square analysis. Correlational analysis was performed initially to exclude variables which were highly correlated with other variables (Spearman's correlation coefficient of 0.7 to 1.0 or – 0.7 to – 1.0) before running the regression model. Odds ratios (OR) and confidence intervals (C.I) obtained from the binary logistic regression analysis were appraised for the likelihood of participants abusing cannabis while controlling for the other variables in the model.

We performed a chi square test to ascertain the association between the likely cannabis abuse and the likelihood of other mental health conditions. There was no data imputation for missing data. The data analyzed and reported reflect the number of responses to each question.

## Results

An online survey link was distributed to 249 residents of Fort McMurray; out of this number, a total of 186 completed the questionnaires with no gross incompletion giving a response rate of 74.7%.

### Descriptive sample characteristics

Table 1 illustrates the demographic profile of the participants and their clinical characteristics examined against relationship status. The prevalence of likely cannabis abuse was 14.0%. The majority were females 159 (85.5%), above the age of 40 years 98 (52.7%), employed, 175 (94.1%), and owed their houses 145 (78.0%). Regarding clinical variables, 78 (41.9%) reported having a history of anxiety diagnosis from a health professional, while 58 (31.2%) reported a history of depression. Again, 59 (31.7%) of the participants were on antidepressants, 21 (11.3%) on sleeping tablets, 72 (38.7%) reported receiving mental health counseling in the past year, and 98 (52.7%) of the participants were willing to receive mental health counseling. Participants, 103 (60.6%) reported experiencing COVID-19 and either Wildfire or flooding traumas.

**Table 1:** Demographic profile, clinical characteristics, and trauma experienced by the study population.

**Variables**	**In a relationship**	**Not in a relationship**	**Total *n* (%)**
	***n* (%)**	***n* (%)**	
**Gender**			
Male	17 (12.9)	10 (18.5)	27 (14.5)
Female	115 (87.1)	44 (81.5)	159 (85.5)
**Age categories**			
≤ 40 y	62 (47.0)	26 (48.1)	88 (47.3)
>40 y	70 (53.0)	28 (51.9)	98 (52.7)
**Employment status**			
Employed	125 (94.7)	50 (92.6)	175 (94.1)
Unemployed	7 (5.3)	4 (07.4)	11 (5.9)
**Housing status**			
Own home	113 (85.6)	32 (59.3)	145 (78.0)
Renting	19 (14.4)	22 (40.7)	41 (22.0)
**History of mental health diagnosis from a health professional?**			
Depression	37 (28.0)	21 (38.9)	58 (31.2)
Anxiety	54 (40.9)	24 (44.4)	78 (41.9)
**History of psychotropic medications**			
Antidepressants	34 (25.8)	25 (46.3)	59 (31.7)
Sleeping tablets	12 (9.1)	9 (16.7)	21 (11.3)
**Respondents received MH counseling in the past year**	48 (36.4)	24 (44.4)	72 (38.7)
**Respondents would like to receive MH counseling**	66 (50.0)	32 (59.3)	98 (52.7)
**Multiple traumas**			
COVID-19 trauma only	13 (10.7%)	6 (12.5%)	19 (11.2)
COVID-19 and either Wildfire or flooding traumas	78 (63.9%)	25 (52.1%)	103 (60.6)
COVID-19, Flooding and Wildfire traumas	31 (25.4%)	17 (35.4%)	48 (28.2)
**No cannabis abuse**	110 (83.3%)	50 (92.6%)	160 (86.0%)
**Cannabis abuse**	22 (16.7%)	4 (7.4%)	26 (14.0%)

MH, mental health.

### Associations between sociodemographic, clinical, and trauma exposure variables and likely cannabis abuse

Table 2 represents the results of the Chi-square analysis. Specifically, seven of the variables were identified *via* Chi-square analysis with significant *p*-values (*p* ≤ 0.05) or *p*-value trending significance (0.1 < *p* < 0.05). The bivariable analysis in Table 2 illustrates statistically significant associations (*p* ≤ 0.05) between gender, housing status, history of depression diagnosis, history of mental health counseling, willingness to receive mental health counseling, and the likelihood of abusing cannabis. In contrast, relationship status and history of antidepressants showed a *p*-value near significance (0.1 < *p* < 0.05).

**Table 2:** Chi-square test of association between cannabis abuse, demographic, and clinical and trauma-related variables.

**Variables**	**No cannabis abuse**	**Cannabis abuse**	**Chi-square**	***P*-value**	**Effect size**
**Gender**
Male	19 (70.4%)	8 (29.6%)	6.434	**0.011**	0.186
Female	141 (88.7%)	18 (11.3%)			
**Age categories**
≤ 40 y	76 (86.4%)	12 (13.6%)	0.016	0.999	0.009
>40 y	84 (85.7%)	14 (14.3%)			
**Employment status**
Employed	152 (86.9%)	23 (13.1%)	1.718	0.367	0.096
Unemployed	8 (72.7%)	3 (27.3%)			
**Relationship status**
In a relationship	110 (83.3%)	22 (16.7%)	2.732	0.098	0.121
Not in a relationship	50 (92.6%)	4 (7.4%)			
**Housing status**
Own home	129 (89.0%)	16 (11.0%)	4.741	**0.029**	0.16
Renting	31 (75.6%)	10 (24.4%)			
**History of depression from a health professional**
Yes	45 (77.6%)	13 (22.4%)	4.987	**0.026**	0.164
No	115 (89.8%)	13 (10.2%)			
**History of anxiety from a health professional**
Yes	65 (83.3%)	13 (16.7%)	0.369	0.369	0.066
No	95 (88.0%)	13 (12.0%)			
**history of antidepressant medications**
Yes	47 (79.7%)	12 (20.3%)	2.907	0.088	0.125
No	113 (89.0%)	14 (11.0%)			
**History of sleeping tablets medications**
Yes	16 (76.2%)	5 (23.8%)	1.903	0.168	0.101
No	144 (87.3%)	21 (12.7%)			
**Respondents received MH counseling** **in the past year**
Yes	57 (79.2%)	15 (20.8%)	4.591	**0.032**	0.157
No	103 (90.4%)	11 (9.6%)			
**Respondents would like to receive MH counseling**
Yes	79 (80.6%)	19 (19.4%)	5.04	**0.025**	0.165
No	81 (92.0%)	7 (8.0%)			
**Multiple traumas**
COVID-19 trauma only	17 (89.5%)	2 (10.5%)	0.192	0.908	0.034
COVID-19 and either wildfire or flooding traumas	89 (86.4%)	14 (13.6%)			
COVID-19, flooding and wildfire traumas	41 (85.4%)	7 (14.6%)			

The bold values indicates the statistically significant values at *p* ≤ 0.05.

Participants who were males, in a relationship, renting accommodation, had a history of depression, received antidepressants, and received mental health counseling in the past, and those who were willing to receive mental health counseling were more likely to abuse cannabis compared to the following, respectively: participants who were females, those who were not in a relationship, staying in their own house, had no history of depression, nor were on antidepressants, no history of receiving mental health counseling and not willing to receive mental health counseling.

### Multivariable binary logistic regression results

Seven of the variables identified *via* Chi-square analysis, as shown in Table 2, with significant *p*-values (*p* < 0.05) or *p*-values approaching significance (0.05 ≤ *p* ≤ 0.1), were illegible for the logistic regression analysis. However, the variables “history of antidepressant medications” was highly correlated (*r* ≥ 0.7) with “History of depression”, hence, not included in the model. As presented in Table 3, the logistic regression model showed the association between independent (demographic and clinical) variables and cannabis abuse among respondents in Fort McMurray. The entire model containing all the six predictors was statistically significant; *X*^2^ (6, *N* = 186) = 28.36, *p* < 0.001, suggesting that the model was able to differentiate between participants who are addicted to cannabis and those who are not addicted to cannabis. The model explained between 14.1% (Cox and Snell *R*^2^) and 25.5% (Nagelkerke *R*^2^) of the variance in the likelihood that participants will present with symptoms of cannabis addiction and correctly classified 86.0% of cases. According to the goodness-of-fit statistic using Hosmer-Lemeshow goodness-of-fit test, the model was adequately fit (*X*^2^ = 5.80; *p* = 0.56).

**Table 3:** Multivariable logistic regression model for participants' likelihood of abusing cannabis.

**Variable**	**Coefficient**	**Standard error**	**Wald**	***P*-value**	**Odds ratio**	**95% C.I for odds ratio**	
						**Lower**	**Upper**
Gender (female)	−1.846	0.614	9.045	**0.003**	0.158	0.047	0.526
Relationship status (not in a relationship)	−1.848	0.681	7.377	**0.007**	0.158	0.041	0.598
Housing status (renting)	1.351	0.541	6.238	**0.013**	3.859	1.337	11.137
History of depression	0.870	0.532	2.671	0.102	2.387	0.841	6.777
Received Mental health counseling in the past	0.238	0.578	0.170	0.680	1.269	0.409	3.939
Would like to receive mental health counseling	1.085	0.643	2.842	0.092	2.959	0.838	10.443

The bold values indicates the statistically significant values at *p* ≤ 0.05. C.I., confidence interval.

Only three of the six predictors made unique contributions to the model (gender, relationship status and housing status). The strongest association (Wald = 9.05) was found among the female gender (OR = 0.158; 95% CI: 0.047–0.526). This suggests that males are six times more likely to abuse cannabis than females. Similarly, participants in a relationship are more than six times more likely to abuse cannabis than participants not in a relationship (OR = 0.158; 95% CI: 0.041–0.598). Finally, participants renting accommodation are approximately four times as likely to abuse cannabis (OR = 3.859; 95% CI: 1.337–11.137).

### Associations between likely cannabis abuse and other mental health conditions

Table 4 shows an association between the likely cannabis abuse and other mental health conditions. The results suggest a significant association between likely moderate to severe depression and cannabis abuse. 14 (18.4%) of the moderate to severe depression participants are likely to abuse cannabis, compared to 7 (7.5%) of those with at most mild depression. Similarly, participants with likely moderate to high anxiety 13 (18.3%) were more likely to abuse cannabis, compared to participants with low anxiety 7 (7.3%).

**Table 4:** Association between the likely cannabis abuse and other mental health conditions.

**Variables**	**No cannabis abuse**	**Cannabis abuse**	**Chi- square/Fisher's Exact**	***P*-value**	**Effect size**
**Likely depression**
At most mild depression	86 (92.5%)	7 (7.5%)	4.561	**0.033**	0.164
Moderate to severe depression	62 (81.6%)	14 (18.4%)			
**Likely suicidal ideations**
Have not had passive death wishes/thoughts of self-harm in the last 2 weeks	125 (89.3%)	15 (10.7%)	2.197	0.138	0.114
Have had passive death wishes/thoughts of self-harm in the last 2 weeks	23 (79.3%)	6 (20.7%)			
**Likely anxiety**
Low anxiety	89 (92.7%)	7 (7.3%)	4.7	**0.03**	0.168
Moderate-to-high anxiety	58 (81.7%)	13 (18.3%)			
**Likely PTSD**
Unlikely PTSD	89 (89.9%)	10 (10.1%)	1.023	0.312	0.079
Likely PTSD	55 (84.6%)	10 (15.4%)			
**Resilience**
High to normal resilience	93 (86.9%)	14 (13.1%)	0.012	0.912	0.008
Low resilience	56 (87.5%)	8 (12.5%)			

## Discussion

The prevalence of self-reported cannabis abuse among residents of Fort McMurray was 14%. After adjusting for confounders, gender, relationship status, and housing status were significantly associated with self-reported cannabis abuse among the study sample. Further, self-reported cannabis abuse was significantly associated with moderate to severe depression and moderate to high anxiety symptoms. Other literature has recorded a varying prevalence of cannabis abuse (2, 8, 44). The variation in prevalence may be attributed to many factors, including the country where cannabis use is legalized. For example, Canada saw an increase in cannabis use since it was legalized and recorded the prevalence of cannabis use at 16.8% in 2019 compared to 14.6% in 2018. On the other hand, a national survey in South Africa recorded 5.0% non-daily and 2.8% daily cannabis use (44). Improper, and continuous use of cannabis use, however, has been associated with other substance use disorders (2). Nonetheless, the majority notice occasional use of cannabis as unremarkable but literature has found that as overall prevalence increases, the risk for cannabis use disorder increases (8).

This study indicates gender as a predictor of likely cannabis abuse, with the prevalence of self- reported cannabis abuse among the male gender being 30% compared to 11% among females. This finding is consistent with several studies which report that males are more likely to use cannabis (2, 44, 45). However, this prevalence of self-reported cannabis abuse is much higher than those reported in a previous study conducted across Canada which reported a prevalence of 1.9% in males and 0.7% in females (45). Males are noted to take an adventure in using cannabis more than females (19), which may account for the increased prevalence of self-reported cannabis abuse in this study which showed that males are six times more likely to use cannabis. On the contrary, another study suggests that females are more likely to abuse cannabis than males once exposed to its use (19). These disparities may be due to genetic variations and social norms (46, 47).

This study also shows a correlation between relationship status and self-reported cannabis abuse. Participants in a relationship are six times more likely to self-report cannabis abuse compared to participants who were not in a relationship. The literature differs on the association between relationship status and cannabis use. One study reported that participants who are not in a relationship have increased stress and may resort to problematic cannabis use (20). A 20-year longitudinal study of cannabis use reported that adult roles and not being married was significantly associated with cannabis use (48). Homish et al. concluded that unmarried people who use cannabis might strongly predict cannabis use in their relationship (49).

This study showed that participants living in rented houses were almost four times more likely to self-report cannabis abuse than those owning their homes. On the contrary, several studies have reported the state of homelessness and substance use. For example, a report on substance usage and homelessness in Canada disclosed that 27.6% of male respondents' substance use is due to housing loss (50). Sekharan also reported that homeless youth and people living in critical housing situations usually use cannabis (22). Whereas, studies associate homelessness with the use of cannabis and its abuse, this study did not collect data specific to homelessness. Thus, we are unable to hypothesize the reason for our findings conclusively. Future research is required to ascertain the relationship between housing status and self-reported cannabis abuse.

Our study found an association between cannabis abuse and other mental health conditions. People experiencing mental illnesses like schizophrenia, anxiety disorders, post-traumatic stress disorder, and depression usually indulge in problematic cannabis use, perceiving that cannabis use is innocuous (9). Comparably, individuals with a history of anxiety or depression may use cannabis to soothe pain or aversive sensations (51). Continuous use of cannabis to manage negative effects associated with mental disorders results in the abuse of the substance (51). More than 18% of the participants in this study reported likely moderate to severe depression symptoms, and self-reported cannabis abuse was positively correlated with presence of moderate to high depression symptoms. The findings from this study are consistent with results from other studies. For example, a study in the United States estimated that cannabis use among people with depression is double the rate of those who are not depressed (11). Major depressive disorder also recorded 19% higher odds in the likelihood of cannabis use in a study conducted by Marmet et al. (52). Similarly, a study to examine risk factors of substance abuse among the youth reported that depressed youths had high odds of abusing cannabis (53). It has also been argued that young people may exhibit suicidal ideations and depressive symptoms when using cannabis (54).

The proportion of participants who reported moderate to severe anxiety symptoms in this study was similar to those who reported moderate to high depression symptoms (18.4%). Anxiety disorder, like other mood disorders, makes an individual vulnerable to the use of cannabis. A meta- analysis concluded that the use of cannabis might cause the onset of affective disorders, including anxiety (12). Predictably, continued use of cannabis is related to elevated prevalence of anxiety disorders and vice versa (55).

Consistent with various literature (17, 31, 54, 56), this study found no significant association between self-reported cannabis abuse and suicidal ideation, PTSD, or low resilience (17, 31, 54, 56). Despite evidence supporting that individual with PTSD use cannabis to mitigate their symptoms (17, 56). Exposure to disasters is relatively common, and individuals may experience at least a trauma in their lifetime; others may develop mental health illnesses like PTSD, substance use disorders, and depression (24, 57) as a result of the trauma. Exposure to traumatic events is usually accompanied by increased cannabis use and abuse (58). Approximately 60.6% of the participants in this study reported experiencing COVID-19 and either wildfire, or flooding. However, this study's traumatic exposure was not significantly associated with self-reported cannabis abuse, which contrasts with the previous study (58). Evidence from the literature suggests the possibility that people with previous experience of serial traumatic events can build resilience as a result of post traumatic adaptation (59). While this phenomenon may potentially partly explain the lack of association found between trauma and self-reported cannabis abuse in Fort McMurray, our recent study suggests that Fort McMurray residents experienced a decline in resilience following these traumatic events (26). Conceivably, such induced low resilience may result in learned helplessness where residents coping mechanisms are diminished (60), including the (ab)use of cannabis or utilization of therapeutic interventions. Perhaps, the latter may explain the high level of depression found among this population in this study. Reflecting further, while the literature reports an association between serial traumatic experiences like multiple terrorist attacks or prolonged conflicts and drug abuse (61), the diverse and unanticipated nature of the multiple disasters (wildfire, flood and epidemic outbreak) experienced by Fort McMurray residents, compared to the sustained or serial occurrence of same trauma, may have implications the differences in responses or coping mechanisms such as substance use. But these are yet untested hypotheses which future studies may explore.

Some studies have compared age and the impact of trauma on substance abuse. Younger individuals experience acute psychological impairments when exposed to traumatic events and are hence more vulnerable than adults to abuse cannabis (62, 63). Yet, this study did not find younger age predictors for cannabis abuse during traumatic exposures. Although studies have shown the effects of traumatic experiences on mental health conditions such as cannabis use disorder, not all people subject to the trauma will develop psychological complications (64).

There are various risks associated with the use of cannabinoids which may be physical, social, or psychological. A systematic review conducted by Giorgetti et al. reported that anxiety, psychosis, cardiovascular, and acute liver, or kidney failure might result from synthetic cannabis usage, which may lead to the death of victims (65). A bi-directional relationship may, however, exist between some of these risks and cannabis use. For example, some studies have viewed mental health challenges as possible risk factors for problematic drug use. A critical review showed that 10–20% of patients with anxiety disorder abuse substances. About 20% of participants in that study agreed that their anxiety problems preceded substance abuse (66). Conversely, some other studies report that mental health problems are more likely to be the adverse effect of problematic substance use than be a predictor (67–71). Thus, cannabinoid use may result from (risk factor) physical and psychosocial challenges or may result in or increase the risk of experiencing these challenges.

## Limitation

Limitations are often unavoidable, especially for studies involving traumatic conditions; therefore, the study's limitations are worth mentioning. Primarily, the sample size for the study was not fully representative of the municipality, given that males are about 5.2% more than females in the municipality and more than 85% of our participants were female, even though they embrace about 47% of the Fort McMurray population. Also, the response rate for the study was computed by using participants who assessed the survey link as the denominator instead of the number who received the survey link, perhaps the overestimation of the response rate. Finally, the scales used to measure likely mental health conditions in the study were self-reported by participants and were not supported by objectivity and detailed clinical assessment. Notwithstanding these limitations, this study is one of the few to examine predictors of likely cannabis abuse. It adds to the literature by documenting potential predictive factors for cannabis abuse and the association with mental health symptoms among a population that has experienced multiple traumas.

## Conclusion

This research highlights potential predictors of likely cannabis abuse among populations who have experienced multiple traumas. These factors include gender, relationship status and housing status. However, the data found no significant association between exposure to trauma and self-reported cannabis abuse. The presence of PTSD symptoms was not associated with self-reported cannabis abuse. However, the presence of moderate to high anxiety and depression symptoms correlated with self-reported cannabis abuse. Further studies are needed in a larger sample to explore the demographic and clinical factors that impact cannabis abuse among populations who have experienced multiple traumas. There is also a need for research into innovative treatment options for individuals presenting with mental health symptoms post-natural disasters to minimize cannabis abuse. Low-cost interventions such as supportive text messaging have been proven effective for supporting the mental health and addiction-related variables among patients (72–76) and the public (77–80) and are geographic location-independent. They can reach thousands of individuals simultaneously with ongoing mental health support post-natural disasters. Finally, policymakers need to mobilize the resources that examine how to create buffer conditions against cannabis abuse.

## Data availability statement

The raw data supporting the conclusions of this article will be made available by the authors, without undue reservation.

## Ethics statement

The studies involving human participants were reviewed and approved by University of Alberta Reviews and Ethics Board. Participants provided consent when they clicked on the survey link and submitted responses.

## Author contributions

VA conceived and designed the study. GO-D drafted the initial manuscript. RS conducted data analysis. EE contributed to data collection. BA reviewed the initial draft manuscript. All authors contributed to the study design, revising the initial draft manuscript, and approving the final draft before submission.

## Funding

This study was supported by grants from the Mental Health Foundation, the Douglas Harden Trust Fund, and Alberta Government. The Fort McMurray Public and Catholic School Boards, Keyano College, and the Canadian Mental Health Association supported survey link distributions. The funders had no role in the design and conduct of the study, collection, management, analysis, the interpretation of the data, preparation, review, or approval of the manuscript, and the decision to submit the results for publication.

## Conflict of interest

Author BA was employed by Global Psychological E-Health Foundation. The remaining authors declare that the research was conducted in the absence of any commercial or financial relationships that could be construed as a potential conflict of interest.

## Publisher's note

All claims expressed in this article are solely those of the authors and do not necessarily represent those of their affiliated organizations, or those of the publisher, the editors and the reviewers. Any product that may be evaluated in this article, or claim that may be made by its manufacturer, is not guaranteed or endorsed by the publisher.
